# The Role of *Allium subhirsutum* L. in the Attenuation of Dermal Wounds by Modulating Oxidative Stress and Inflammation in *Wistar* Albino Rats

**DOI:** 10.3390/molecules26164875

**Published:** 2021-08-12

**Authors:** Mongi Saoudi, Riadh Badraoui, Ahlem Chira, Mohd Saeed, Nouha Bouali, Salem Elkahoui, Jahoor M. Alam, Choumous Kallel, Abdelfattah El Feki

**Affiliations:** 1 Animal Ecophysiology Laboratory, Sciences Faculty of Sfax, University of Sfax, Sfax 3054, Tunisia; chiraahlem@gmail.com (A.C.); abdelfattahelfeki@fss.rnu.tn (A.E.F.); 2 Laboratory of General Biology, Department of Biology, University of Ha’il, Ha’il 81451, Saudi Arabia; mo.saeed@uoh.edu.sa (M.S.); nouha_bmail@yahoo.fr (N.B.); s.elkahoui@uoh.edu.sa (S.E.); j.alam@uoh.edu.sa (J.M.A.); 3 Section of Histology and Cytology, Medicine Faculty of Tunis, University of Tunis El Manar, La Rabta, Tunis 1007, Tunisia; 4 Hematology Laboratory, Hospital Habib Bourguiba, Sfax 3029, Tunisia; kallelC@yahoo.fr

**Keywords:** *Allium subhirsutum* L., wound-healing activity, antioxidant potential, inflammatory marker, oxidative stress

## Abstract

In our study, *Allium subhirsutum* L. (AS) was investigated to assess its phenolic profile and bioactive molecules including flavonoids and organosulfur compounds. The antioxidant potential of AS and wound healing activity were addressed using skin wound healing and oxidative stress and inflammation marker estimation in rat models. Phytochemical and antiradical activities of AS extract (ASE) and oil (ASO) were studied. The rats were randomly assigned to four groups: group I served as a control and was treated with simple ointment base, group II was treated with ASE ointment, group III was treated with ASO ointment and group IV (reference group; Ref) was treated with a reference drug “Cytolcentella^®^ cream”. Phytochemical screening showed that total phenols (215 ± 3.5 mg GAE/g) and flavonoids (172.4 ± 3.1 mg QE/g) were higher in the ASO than the ASE group. The results of the antioxidant properties showed that ASO exhibited the highest DPPH free radical scavenging potential (IC50 = 0.136 ± 0.07 mg/mL), FRAP test (IC50 = 0.013 ± 0.006 mg/mL), ABTS test (IC50 = 0.52 ± 0.03 mg/mL) and total antioxidant capacity (IC50 = 0.34 ± 0.06 mg/mL). In the wound healing study, topical application of ASO performed the fastest wound-repairing process estimated by a chromatic study, percentage wound closure, fibrinogen level and oxidative damage status, as compared to ASE, the Cytolcentella reference drug and the untreated rats. The use of AS extract and oil were also associated with the attenuation of oxidative stress damage in the wound-healing treated rats. Overall, the results provided that AS, particularly ASO, has a potential medicinal value to act as effective skin wound healing agent.

## 1. Introduction

Wound healing is a complicated and dynamic physiological process in response to tissue impairment [1]. It is a fundamental connective tissue response [2] to tissue impairment through three phases, including hemostasis and inflammation, proliferation and remodeling [3]. Cutaneous wound healing involves several mediators, such as fibroblasts, endothelial cells, blood cells, interactions with extracellular phases and granulation tissue remodeling phases [4,5]. Furthermore, reactive oxygen species (ROS) are produced in response to cutaneous injury, and act as cellular messengers to stimulate several physiological processes, such as cytokine action, cell motility and angiogenesis [4]. However, cutaneous injury affects the healing process by the overproduction of ROS and the perturbation of various enzymatic and non-enzymatic antioxidant defenses [6], particularly during the inflammatory phase [7]. Long-term instability and high concentration of ROS may cause cellular injury by damaging both proteins and membrane lipids, the perturbation of antioxidant enzymes and the breakdown of the nucleic acids, particularly DNA [8,9]. Higher concentrations of intracellular ROS might eventually lead to angiogenesis pathological damage, promoting inflammation [10,11], which makes blood flow and nutritional requirements unable to meet the needs of wound healing [12].

Many antioxidants in medicinal plant extracts are used to eliminate the negative effects of ROS-associated pathologies and/or injuries including wound healing [11,13,14]. Over the years, medicinal plants have been used to develop a variety of formulations that combat injuries such as wounds, burns and cuts [15]. Several ethno-medicinal plants have been used for medicinal applications thanks to their wide biological activities and medicinal applications due to counteracting oxidative stress potential, lesser costs and high safety margins [16,17,18]. Plant extracts are known to exhibit many pharmacological properties (anti-hyperlipidemic, anti-proliferative, and immunomodulatory, etc.) through the multitude amounts of natural polyphenolic antioxidants [17,18,19].

Garlic species are used as dietary supplements and an important ingredient for cooking in many parts of the world and their application in medicinal remedy has also increased its popularity [11,17,20]. Garlic species are known to have diverse biological activities, particularly due to their antioxidant properties. *Allium subhirsutum* L. is an aromatic plant known since antiquity. It was investigated in terms of its potential antioxidant components such as flavonoids and polyphenols [10,11,21]. Previous studies reported that it is a potential source of anticancer and antioxidant molecules [16]. It is a very rich source of valuable compounds, such as polyphenols, vitamins, flavonoids, carotenoids and carbohydrates [17]. Experimental studies showed that garlic phenolic compounds and flavonoids may promote epithelialization and stimulate new tissue growth, fluid handling and moist wound healing [22]. As regards to its pharmacological properties, garlic has been revealed to display antidiabetic, hepatoprotective, antimicrobial, renoprotective, immunomodulatory and anti-inflammatory properties [23].

Furthermore, garlic is also rich in different bioactive compounds like allicin, glutathione diallyl sulfides [24] and important minerals (selenium, manganese and zinc) which encourage its consumption. Pre-treatment with garlic significantly reduced levels of ROS, lipid peroxidation and DNA damage, and thus enhanced the antioxidant system [11,25]. Recent studies [26] revealed that the application of bioactive molecules with antioxidant activities improved wound healing and protected against oxidative damage. Wound care medicinal plants in the form of ointment could ameliorate wound healing and tissue repair with minimal side effects [27]. Previous studies reported that infections in the locations of injured skin are the main reasons for mortality in hospitalized patients with extensive burns [28]. For that reason, the application of plant medicinal preparations as local ointment with considerable antimicrobial effects can significantly reduce the risk of burn wound infections and alleviate the period of treatment [29]. Garlic has been reported to accelerate wound contraction rate, validated by a significant improvement and an increase in the rate of wound closure and a reduction in the time taken by the granulation tissue and inflammatory marker levels to fall [30].

With these considerations, the current study aimed to evaluate wound healing activity of AS extract and oil, using a skin wound healing rat model via the estimation of oxidative stress and anti-inflammatory parameters. This research on the antioxidants and in vivo biological activities of AS can improve its commercial value and help to develop new nutritional and health products.

## 2. Results & Discussion

### 2.1. Phytochemical Analysis

In the current study, the phytochemical analysis of *Allium subhirsutum* L. extract and oil showed their richness in phenolic and flavonoid compounds. Furthermore, higher contents of polyphenols and flavonoids confirmed the presence of these biomolecules in ASO.

Total polyphenols, flavonoids and tannins of *Allium subhirsutum* L. extract and oil were calculated according to the calibration curves and the phytochemical amounts are shown in Table 1. Phytochemical screening showed that total polyphenol (215 ± 3.5 mg GAE/g) and flavonoid (172.4 ± 3.1 mg QE/g) amounts are higher in the ASO than the ASE. However, the total tannin level was higher (387.5 ± 17.2 mg QE/g) in the ASE than ASO, in which it was not detected. Total polyphenols, flavonoids and tannins of *Allium subhirsutum* L. extract and oil were calculated according to the calibration curves (see Supplementary Figure S1). The richness in phenolic and flavonoid compounds have been highlighted in *Allium subhirsutum* by previous studies, including those realized by our team [11,17]. Nevertheless, the variations in such compounds might be related to the region of the plant collection.

### 2.2. Antioxidant Potential Analysis

The antioxidant properties of the extract and oil of *Allium subhirsutum* L. were assessed via several approaches: scavenging activity on DPPH free radicals, reducing power, total antioxidant activity assay by ABTS and total antioxidant capacity. The results are summarized in Table 1. The antioxidant activities of the studied *Allium subhirsutum* L. extract and oil were compared with vitamin C as a standard antioxidant. The *Allium subhirsutum* L. oil exhibited the highest DPPH free radical scavenging potential (IC50 = 0.136 ± 0.07 mg/mL), FRAP test (IC50 = 0.013 ± 0.006 mg/mL), ABTS test (IC50 = 0.52 ± 0.03 mg/mL) and total antioxidant capacity (IC50 = 0.34 ± 0.06 mg/mL), as compared to *Allium subhirsutum* L. extract. The results revealed an important difference of the DPPH, FRAP, ABTS and total antioxidant capacity tests between the standard vitamin C and the different extracts. Furthermore, the *Allium subhirsutum* L. extract and oil showed potential antioxidant activities as compared to vitamin C; thus, it is able to scavenge superoxide and peroxyl radicals.

Antioxidant activities as estimated by DPPH free radicals, reducing power, total antioxidant activity assay and total antioxidant capacities in ASO explain the ROS scavenging capacity, but it was less than the standard vitamin C. The antioxidant status of ASO is related to the major active components such as organosulfur molecules and their derivatives. Many garlic supplements such as garlic oil macerate, garlic oil, dehydrated garlic powder and aged garlic extract are currently commercially available [31].

### 2.3. Wound Closure

#### 2.3.1. Chromatic Study

Considering the medicinal application of *Allium subhirsutum* L. and the in vitro antioxidant activity, the plant was further evaluated for skin lesion potential, particularly burns and wounds. Several garlic species have been evaluated in different animal models and were reported to display cutaneous wound healing [32].

**Figure 1:**
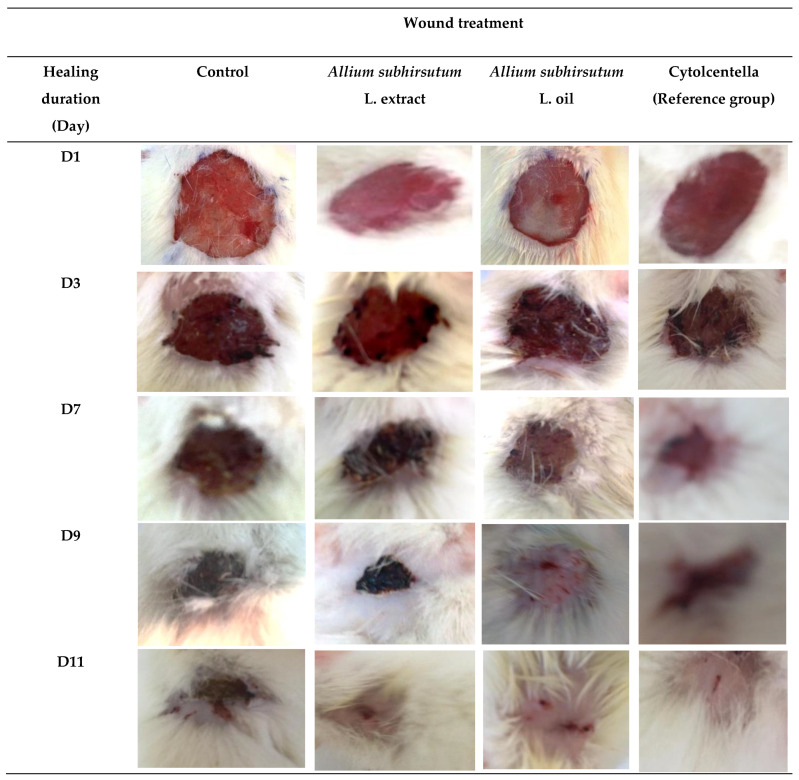
Photographic illustrations of wound healing process in the different experimental groups on 1, 3, 7, 9 and 11 days post-wounding.

The wound healing activity was checked by a chromatic assessment based on the progressive variations of both wound color and surface during the experimental study. The different phases of cicatrization of all studied groups are shown in Figure 1.

The selected days (1, 3, 7, 9 and 11) correspond to the wound induction day, the epithelialization progress and the inflammatory evolution. The wounds’ photographic representations denoted a similar surface and colored wound areas in the first three days for the different studied groups of rats. The photographs of the wound revealed a bright red color during the first day that turned dark red on the third day, which proves blood clot formation. By the seventh day of wound healing, a large inflammatory bulb in the control group was noted, while the wound surfaces of *Allium subhirsutum* L. extract and oil-treated and Cytolcentella-treated rats were smaller. From the ninth day, in the rats treated with ASO and Cytolcentella (Ref group), the crusts began to fall off revealing a red granulated tissue, which characterized completed epithelialization around the 11th day. The process was exhibited at the eleventh day to the complete wound closure in the ASO and Cytolcentella reference drug groups. However, the *Allium subhirsutum* L. extract and control group wounds showed residual scabs and healed slower. The closure was uncompleted in the control and *Allium subhirsutum* L. extract-treated groups.

The wound photographic representations of the treated groups ASO and Cytolcentella reference drug showed better and more advanced epithelial regeneration when compared to the control group (group I). This faster wound closure of *Allium subhirsutum* L. oil, as compared to the ointment by Cytolcentella reference drug, could presumably be due to the richness of *Allium subhirsutum* L., particularly polyphenols and flavonoids, and with several other bioactive compounds (sulfur-rich compounds), as demonstrated by Badraoui et al. [17] Previous findings [11,17,33] revealed that polyphenols play a key role in the proliferation of epithelial cells and the regulation of angiogenesis.

#### 2.3.2. Effect of *Allium Subhirsutum* L. Extract and Oil on Percentage Wound Closure

**Figure 2:**
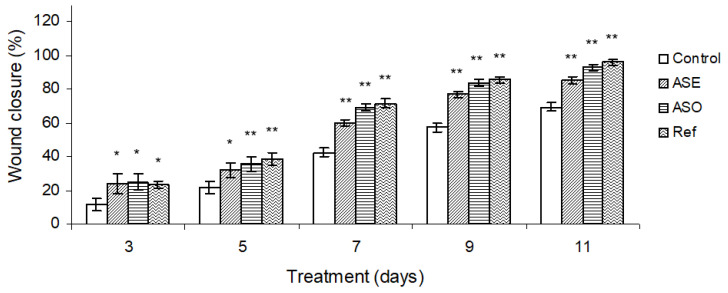
Effects of *Allium subhirsutum* L. extract (ASE), *Allium subhirsutum* L. oil and the reference drug “Cytolcentella^®^” on the percentage of wound closure in control and treated rats. Data represent the mean ± SEM for six rats. Statistically significant variations are compared as follows: ASE, ASO and Ref treated groups compared to control group. * and ** indicate significant differences when *p* < 0.05 and *p* < 0.01, respectively.

The changes in the percentage of wound closure in control, *Allium subhirsutum* L. extract and oil, and the reference drug “Cytolcentella” groups were followed on some selected days (Figure 2).

The *Allium subhirsutum* L. extract and oil group displayed a marked wound-healing potential as compared to the untreated group and the Cytolcentella reference drug group. However, the *Allium subhirsutum* L. extract showed a lower wound-healing process as compared to the *Allium subhirsutum* L. oil and Cytolcentella reference drug groups. On days 9 and 10, a rapid reduction in wound area of the *Allium subhirsutum* L. oil group was observed (*p* < 0.01), which was comparable to the Cytolcentella reference drug group.

The reference group showed the strongest wound contraction rate throughout healing days and complete healing of the wound (100%) was observed on the 11th day, while the *Allium subhirsutum* L. extract and oil group showed 81.25% and 87% healing on same day, respectively. It was also noticed that no comparable wound area evolution was observed between the ASO, ASE and Cytolcentella groups on the first 3 days.

Additionally, the morphometric assessment exhibited an increased wound contraction rate of the wounds following ASO and the Cytolcentella reference drug treatment as compared to the untreated group. Previous research using an aqueous extract of garlic (*Allium sativum*) showed a higher percentage of burn wound contraction in rats treated with 0.4% garlic topical cream (88.1%) than those treated with cream base (70.3%) [28]. This rapid wound closure may be attributed to an increase in fibroblast activity, which is crucial for normal wound contraction.

#### 2.3.3. Effect on Inflammatory Marker

**Figure 3:**
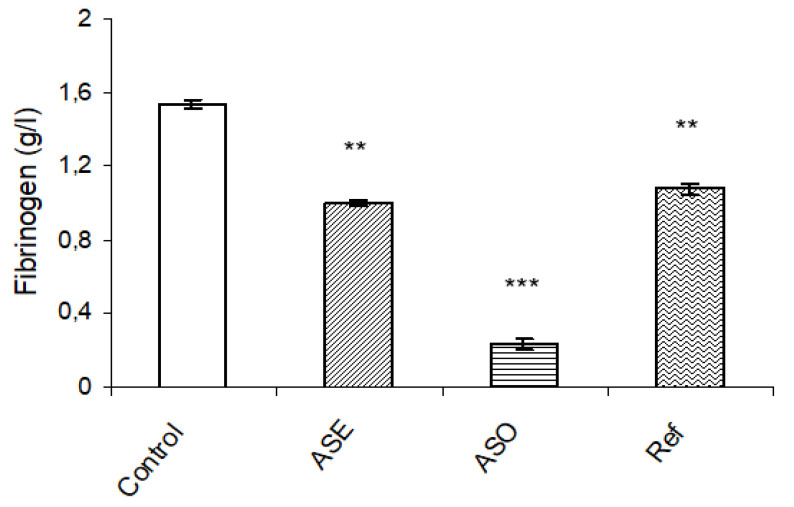
Effects of *Allium subhirsutum* L. extract (ASE), *Allium subhirsutum* L. oil and the reference drug “Cytolcentella^®^” on fibrinogen level. Data represent the mean ± SEM for six rats. Statistically significant variations are compared as follows: ASE, ASO and Ref treated groups compared to control group: ** and *** indicate significant differences when *p* < 0.01 and *p* < 0.001, respectively.

The inflammatory marker of different groups was analyzed to determine the level of fibrinogen (Figure 3), which is known as an inflammatory protein. An increased level of this protein revealed a condition of inflammation. Fibrinogen content was found to be significantly alleviated in *Allium subhirsutum* L. oil (*p* < 0.001) and the Cytolcentella reference drug (*p* < 0.01) groups, followed by *Allium subhirsutum* L. extract (*p* < 0.01) as compared to control group.

The results confirmed reduced levels of the inflammatory marker (fibrinogen content) on topical treatments with ASO and ASE as compared to the control group and the reference drug Cytolcentella group. The present study corroborates previous findings [11,34], which notified that phenolic compounds have the potency to attenuate the inflammatory markers as well as pro-inflammatory cytokines, which accelerate the mechanism of collagenation and maturation of granulation tissue.

### 2.4. Oxidative Stress Profile

Recent findings have shown that oxidative stress can cause cell damage [9,35,36,37] and delay wound healing [38]. The formation of free radicals and a low capacity to scavenge ROS are causative of skin lesions. After an overproduction of ROS, an increase in oxidative stress markers, as estimated by lipid peroxidation and protein oxidation molecules resulted in the healing process delay. The attenuation of excessive ROS generation significantly accelerates the healing process. The cells respond by developing a defense mechanism that consists of producing antioxidant enzymes, including superoxide dismutase (SOD), catalase (CAT) and glutathione peroxidase (GPx).

#### 2.4.1. Oxidative Stress Markers of Granulation Tissue

The levels of wound tissues TBARS, CD, AOPP and CP are shown in Table 2. Results indicated that untreated control rats possessed the highest oxidative stress markers, proving the presence of an oxidative stress state in these rats. However, the application of either *Allium subhirsutum* L. extract and oil or the reference drug Cytolcentella significantly decreased the levels of TBARS, CD, AOPP and CP in the wound tissues as compared to control.

In our experiment, the treatment with ASO and ASE significantly decreased wound tissue levels TBARS, CD, AOPP and CP as compared to control rats. These results are in accordance with previous results [11,12,13,14,17,39], which reported that plants, specifically hairy garlic, possessed high antioxidant and free radical-scavenging effects. It may be attributed to the presence of active biomolecules in *Allium subhirsutum* L. oil which ultimately cause an antioxidant activity and wound repairing. The availability of bioactive molecules such polyphenols and flavonoids, and the ROS-scavenging ability of *Allium subhirsutum* L. helped in lowering the ROS levels, thus accelerating the wound closure.

#### 2.4.2. Enzymatic Antioxidant Profile of Granulation Tissue

Several studies have shown that enzymatic antioxidant profiles such as SOD, CAT and GPx scavenge free radicals and prevent oxidative damage.

Antioxidant enzyme profiles (SOD, CAT and GPx) of control and treated rats are summarized in Table 3. The tissue antioxidant enzymatic activities of the control group were the lowest. The pre-treatment with *Allium subhirsutum* L. and the reference drug Cytolcentella showed a significant increase in the enzymatic activities as compared to the control group.

In the current study, we observed an increase in SOD, CAT and GPx activities in the wound area in rats treated with ASO, ASE and the reference drug as compared to control rats. These results revealed that topical application of *Allium subhirsutum* L. oil and extract protected against cellular damage by the stimulation and/or the expression of the antioxidant enzymes. Our findings are in accordance with the data authored by Khan et al. [23], in which they reported that *Allium sativum* oil protected against oxidative damage in fish exposed to silver nanoparticles (Ag-NPs), and with Zammel et al. [11], where *Allium subhirsutum* was found to protect against carrageenan-induced paw edema, inflammation and oxidative damage in rats.

#### 2.4.3. Correlation Matrix between Phytochemicals of *Allium subhirsutum* L., Oxidative Stress, Fibrinogen and Wound Reduction

Table 4 presented the correlation matrix between the antioxidant equivalent markers (polyphenols, flavonoids, tannins), the oxidative/antioxidative status, the fibrinogen level and the percentage of wound reduction (on the 11th day of treatment). As shown in Table 4, this correlation matrix revealed that AOPP oxidative stress marker, SOD and CAT enzymatic antioxidant activities were positively correlated with the antioxidant equivalent marker in *Allium subhirsutum* extract and oil (polyphenols, flavonoids and tannins), indicating its involvement in the attenuation of oxidative stress damage, as previously confirmed by several other reports [11,40].

*Allium subhirsutum* oil protected against oxidative damage as demonstrated by the SOD and GPx activities which were positively correlated with the polyphenols and flavonoids contents of ASO. A similar positive correlation was previously observed in *Lonicera caerulea* L. polyphenols that alleviated oxidative stress-induced intestinal environment imbalance and lipopolysaccharide-induced liver injury in high-fat diet-fed rats [41].

## 3. Materials and Methods

### 3.1. Plant Material and Extraction

Fresh plant *Allium subhirsutum* L. cloves was procured from a local market in the Sfax region, Tunisia in October 2020. The plant specimen was washed under running water and skins of the samples were removed. For the extract preparation, *Allium subhirsutum* L. cloves were extracted and stirred with methanol at 30 °C for one night. Then, a whatmann filter paper was used to remove the particles. The residue was once more extracted, filtered, concentrated and stored until further use. The oil extraction was determined using the application of pressure as described by the method of Arisanu and Rus [42]. By this method, oil is enhanced by increased mechanical pressure on the oil-bearing material.

### 3.2. Phytochemical Analysis

#### 3.2.1. Total Phenolic Content

The total phenolic content of the *Allium subhirsutum* L. extract and oil was measured using a modified colorimetric Folin–Ciocalteu method [43]. The total phenolic content was expressed as mg of gallic acid equivalents (GAE) per gram of dry weight.

#### 3.2.2. Total Flavonoid Content

The total flavonoid content was determined by a colorimetric assay using the aluminum trichloride method according to Chang et al. [44]. Flavonoid content was expressed as mg of quercetin equivalent (QE)/gram of extract.

#### 3.2.3. Total Tannins Content

The total tannins content of *Allium subhirsutum* L. extract and oil were determined as described by Broadhurst et al. [45] 50 µL of the extract was mixed with vanillin/methanol (3 mL, 4%). After stirring, 1.5 mL concentrated HCl was added. After 15 min, the absorbance was measured at 500 nm and the total tannins content was expressed as mg quercetin equivalent (QE)/gram of extract.

### 3.3. Antioxidant Potential Analysis

#### 3.3.1. DPPH Free Radical Scavenging Assay

The DPPH• (2,2-diphenyl-1-picrylhydrazyl) free radical scavenging activities of *Allium subhirsutum* L. extract and oil were determined using the stable radical 1.1-diphenyl-2-picrylhydrazyl (DPPH), as previously reported by Kirby and Schmidt [46]. A total of 1 mL of different extract concentrations (0.06–1.0 mg × mL^−1^) in methanol was mixed with 1 mL of DPPH radical solution in methanol 4% (*w/v*).

The absorbance of the samples and control solutions were measured at 517 nm against a blank. The antiradical activity was expressed as IC50 (µg/mL). The inhibition was calculated as follows:

Free radical DPPH inhibition (%) = 100 × (A_control_ − A_sample_)/A_control_

where A_control_ is the absorbance of the control reaction (without the test compound) and A_sample_ is the absorbance of the test compound. Ascorbic acid was used as a control.

#### 3.3.2. Ferric Reducing Antioxidant Power

The reducing power was assessed using the method described by Oyaizu [47]. *Allium subhirsutum* L. extract and oil (0.06–1.0 mg × mL^−1^) was mixed with 1 mL of sodium phosphate buffer (200 mM, pH 6.6) and 1 mL of potassium ferricyanide (1%, K_3_Fe(CN)_6_). After incubation at 50 °C for 20 min, 1 mL of trichloroacetic acid (10%) was added. The mixture was then centrifuged for 10 min at 650× *g*. An amount of 1.5 mL of the supernatant was mixed with 0.1 mL of ferric chloride (0.1%, FeCl_3_) and 1.5 mL of deionized water. The absorbance was read at 700 nm and compared to a blank. The IC50 value (mg × mL^−1^) was determined and compared to the ascorbic acid activity, which was used as standard.

#### 3.3.3. Total Antioxidant Activity Assay by Radical Cation (ABTS+)

The Trolox equivalent antioxidant capacity (TEAC) determines the reduction in 2,2′-azino-bis(3-ethylbenzothiazoline-6-sulphonic acid, as previously reported by Re et al. [48] The antioxidant activity was expressed as mg TE/g extract and the IC50 was determined.

#### 3.3.4. Total Antioxidant Capacities (TAC)

TAC of the plant substances (extract and oil) were assessed by the phosphomolybdenum method as described by Prieto et al. [49] Briefly, 0.1 mL of methanolic sample solution was mixed with 1 mL of the reagent solution (0.6 M, 28 mM and 4 mM of sulfuric acid, sodium phosphate and ammonium molybdate, respectively) and incubated in a boiling water bath for 90 min at 95 °C. After cooling at room temperature, the absorbance was determined at 695 nm and compared to a bank.

### 3.4. Wound Healing Assay

Due to its anti-inflammatory and antioxidant potentials, *Allium subhirsutum* L. extract and oil were tested for their wound healing in rats.

#### 3.4.1. Animals

A total of 24 male albino *Wistar* rats weighing between 230–270 g were used for this study. The rats were purchased from the Faculty of Sciences of Gabes, Tunisia. The rats were allowed to acclimatize to the housing conditions (22 ± 3 °C, 12 h light/dark cycles and about 42% humidity). The rats were given a standard diet (SICO, Sfax, Tunisia) and ad libitum. All experiments involving animals were conducted according to the Ethical Committee Guidelines for the care and use of laboratory animals of our institution (University of Sfax, Tunisia) and approved by the local committee.

#### 3.4.2. Wound Treatment

After anesthesia (intraperitoneal injection of chloral hydrate), an impression by a round seal of 1 cm diameter was made on the dorsal thoracic regions of the rats. The skin of the impressed area (sterilized and shaved before circular wound creation) was superficially excised to the full thickness to create the wound area [50].

Wounds rats were randomly divided into 4 groups of 6 rats each. Group I was treated with sterile saline and designated as negative control. Group II was treated with *Allium subhirsutum* L. extract (ASE), group III was treated with *Allium subhirsutum* L. oil (ASO), and group IV (reference group) was treated with a reference drug “Cytolcentella^®^” (Ref).

After cleaning the wounds with sterile saline, all treatments using extract and oil of *Allium subhirsutum* L. and the reference drug “Cytolcentella^®^” were topically applied every two days until sacrifice.

#### 3.4.3. Percentage of Wound Closure Rate

The wound perimeters were traced using transparent paper and the percentage of the healed area was recorded. The percentage of wound closure was calculated using the following formula [51]:

Wound closure (%) = (Area of wound on day 0 − Area of wound on nth day)/(area of wound on day 0) × 100

where n represents the number of days, i.e., 3rd, 5th, 7th, 9th and 11th.

The required number of days for complete wound contraction was registered together with the re-epithelialization progress and the infection evolution were evaluated using macroscopic images.

#### 3.4.4. Collection of Blood and Tissue

After sacrifice under chloral hydrate anesthesia, blood samples were collected by cardiac puncture and centrifuged at 2700× *g* for 15 min for various biochemical parameters, such as fribrinogen markers. The skin samples were collected, and the dissected wound tissue was used for analysis of oxidative damage markers and the enzymatic antioxidant defense system. For oxidative stress profile studies, each collected wound tissue was homogenized in TBS buffer (Tris-HCl 50 mM and NaCl 150 mM (1:2, *w*/*v*)) at pH 7.4 using an Ultra-Turax homogenizer. After centrifugation at 10,000× *g* for 15 min at 4°C, the supernatant was used to determine the protein content, as described by Lowry et al. [52]

#### 3.4.5. Determination of Inflammatory Markers

Inflammatory markers of treated and control rats were evaluated after the sacrifice by measuring fibrinogen levels in blood samples. The level of fibrinogen was measured using a commercial kit obtained from STA Liquid fibrinogen (diagnostica stago), according to the manufacturer’s instructions.

### 3.5. Determination of Oxidative Stress Markers of Granulation Tissue

#### 3.5.1. Thiobarbituric Acid Reactive Substances (TBARS)

TBARS was assessed after the sacrifice using the spectrophotometric method described by Buege and Aust [53]. A total of 375 μL of extract, 150 μL of TBS and 375 μL of TCA 20% and 1% BHT were mixed to deproteinize the extracts. After centrifugation (1000× *g* for 10 min), 400 μL of the supernatant were mixed with 80 μL of HCl (0.6 M) and 320 μL of Tris-TBA (26 mMTris, 120 mM thiobarbituric acid). The optical density was measured at 530 nm. The TBARS amount was measured using 0.156 mM^−1^ cm^−1^ as the extinction coefficient.

#### 3.5.2. Conjugated Diene (CD)

Conjugated diene levels were evaluated after the sacrifice by Halliwell and Gutteridge [54]. A total of 25 μL of wound tissue, 3 mL of chloroform and methanol (2:1, *v*/*v*) were mixed and then centrifuged at 3000 rpm for 5 min. An amount of 2 mL of the supernatant were taken and dried at 45 °C overnight. The extract obtained is again dissolved in 2 mL of methanol. We read the OD at 190 nm.

#### 3.5.3. Advanced Oxidation of Protein Products Levels (AOPP)

The AOPP level was assessed after the sacrifice using the spectrophotometric method described by Witko et al. [55] Amounts of 200 μL of potassium iodide and 20 μL of acetic acid were placed in the presence of the sample diluted using 800 μL of PBS. AOPP has the capacity to absorb at 340 nm in an acid medium. Chloramine-T (0 to 200 μM) used as standard absorbed at 340 nm in the presence of potassium iodide. 

#### 3.5.4. Carbonyl Protein (CP)

The CP was assessed after the sacrifice using the spectrophotometric method described by Reznick and Packer [56]. A solution of TCA (20%) was added to the samples of the homogenate in order to precipitate the proteins. Then, a solution of DNPH (10 mM) was solubilized in HCl (2N) for 1 h at room temperature. The mixture was centrifuged for 15 min at 4 °C at 4000× *g*. The samples were treated with a guanidine–HCl solution (6 M) and placed in water at a temperature of 37 °C for 15 min. The protein carbonyl content was measured at 370 nm against a guanidine blank using a molar extinction coefficient of 22,000 M 1 cm^−1^.

### 3.6. Determination of Enzymatic Antioxidant Profile of the Granulation Tissue

#### 3.6.1. Superoxide Dismutase Activity (SOD)

SOD activity was assessed after the sacrifice using the spectrophotometric method described by Beyer and Fridovich [57]. The reaction mixture containing 50 mM of the tissue homogenates was prepared; then, the absorbance was measured at 560 nm and the SOD activity was expressed as unit per mg of protein (Unit/mg protein).

#### 3.6.2. Glutathione Peroxidase Activity (GPx)

GPx activity was assessed after the sacrifice using the method described by Flohe and Gunzler [58] using 5% TCA. After centrifugation at 1500× *g* for 10 min, the supernatant was collected. A total of 0.1 mL of the tissue supernatant was mixed with 0.7 mL of 5.50 dithiobis-(2-nitrobenzoic acid) and (DTNB, 0.4 mg/mL) and 0.2 mL of phosphate buffer (0.1 M pH 7.4). The absorbance was measured at 420 nm and the GPx activity was expressed as nmoles of GSH/min/mg protein.

#### 3.6.3. Catalase Activity (CAT)

CAT activity was assessed after the sacrifice using the method described by Aebi [59]. Briefly, 20 µL of tissue homogenate was added to 880 µL of H_2_O_2_ solution (pH = 7.4), which contains 0.5 mol/L H_2_O_2_ and 0.1 mol/L of phosphate buffer. The principle of the method is based on monitoring the H_2_O_2_ decomposition spectrophotometrically at 290 nm via the absorbance decrease. The extinction coefficient was 0.043/mM^−1^cm^−1^ and the enzyme activity was expressed as µmol H_2_O_2_ decomposed/min/mg of protein (µM/min/mg protein).

### 3.7. Statistical Analysis

All results were expressed as mean ± standard error of the mean (SEM). Differences were considered statistically significant at *p* ≤ 0.05. The results were analyzed by one-way analysis of variance (ANOVA) to assess the comparisons between groups using SPSS for Windows (version 18).

## 4. Conclusions

In conclusion both ASE and ASO possessed interesting amounts of polyphenols. Nevertheless, ASO was shown to contain more flavonoids and polyphenols. These high levels were associated with good antioxidant potential as assessed by DPPH, FRAP, ABTS and total antioxidant capacity and experimental wound healing in rats. They were also associated with better activity in terms of inflammation biomarkers, antioxidant parameters and wound healing activity. The latter revealed significantly accelerated both re-epithelialization and vascularization processes. The polyphenol, flavonoid and tannin contents of AS paralleled the positive effects on wound healing and the inflammatory reduction in the wound-healing treated rats. These results confirmed the ethno-pharmacological potential of *Allium subhirsutum* and encourage its use in the pharmaceutical industry due to its promising activity, particularly the oil fraction.

## Figures and Tables

**Table 1:** Amounts of total phenolic components, flavonoids, tannins and IC50 for the DPPH scavenging activity, ferric reducing antioxidant power (FRAP), ABTS antioxidant activity and total antioxidant capacities (TAC) of *Allium subhirsutum* L. extract and oil. Ascorbic acid was used as standard.

Samples and Parameters	Total Polyphenols ^a^ (mg GAE/g) ^b^	Flavonoids ^a^ (mg EQ/g) ^c^	Tannins ^a^ (mg EQ/g)	DPPH ^a^ IC50 (mg/mL)	FRAP ^a^ IC50 (mg/mL)	ABTS ^a^ IC50 (mg/mL)	Total Antioxidant Capacity ^a^ (mg VitC/g) ^d^
***A. subhirsutum* L. extract**	63.8 ± 2.36	41.7 ± 3.4	387.5 ± 17.2	0.20 ± 0.004	0.05 ± 0.004	0.54 ± 0.04	0.45 ± 0.09
***A. subhirsutum* L. oil**	215 ± 3.5	172.4 ± 3.1	ND	0.136 ± 0.07	0.013 ± 0.006	0.52 ± 0.03	0.34 ± 0.06
**Ascorbic acid**	—	—	—	0.118 ± 0.006	0.08 ± 0.004	0.09 ± 0.09	0.124 ± 0.002

^a^: Data represent the mean ± SEM of three experiments; ^b^: mg of gallic acid equivalent/g of dry plant extract; ^c^: mg of quercetin equivalent/g of dry plant extract; ^d^: mg Vitamin C equivalent/g of dry plant extract; ND: not detected; —: not tested.

**Table 2:** Effects of *Allium subhirsutum* L. extract (ASE), *Allium subhirsutum* L. oil and the reference drug “Cytolcentella^®^” on oxidative stress markers of granulation tissue by estimation of TBARS, CD, AOPPA and CP levels.

Treatment & Parameters	TBARS (nmol MDA/mg Protein)	CD (µmol/mg Protein)	AOPP (µmol/mg Protein)	CP (µmol/mg Protein)
**Control**	1.38 ± 0.138	0.69 ± 0.04	0.25 ± 0.01	61.37 ± 0.37
**ASE**	1.08 ± 0.09 *	0.58 ± 0.13 *	0.23 ± 0.003	50.73 ± 1.28 **
**ASO**	0.91 ± 0.37 *	0.48 ± 0.11 **	0.21 ± 0.003 *	47.63 ± 1.09 **
**Ref**	0.82 ± 0.014 **	0.45 ± 0.01 ***	0.20 ± 0.006 **	41.80 ± 1.14 ***

The oxidative stress marker parameters: TBARS: Thiobarbituric acid-reactive substances; CD: conjugated dienes; AOPP: advanced oxidation of protein products; CP: carbonyl protein. Data represent the mean ± SEM for six rats. Statistically significant variations are compared as follows: ASE, ASO and Ref treated groups compared to control group. *, ** and *** indicate significant differences when *p* < 0.05, *p* < 0.01 and *p* < 0.001, respectively.

**Table 3:** Effects of *Allium subhirsutum* L. extract (ASE), *Allium subhirsutum* L. oil and the reference drug “Cytolcentella^®^” on enzymatic antioxidant profile of granulation tissue by estimation of SOD, CAT and GPx activities.

Treatment & Parameters	SOD (Units/mg Protein)	CAT (µmol H_2_O_2_/mg Protein)	GPx (µmol GSH/min/mg Protein)
**Control**	18.47 ± 0.29	50.50 ± 1.18	0.009 ± 0.0006
**ASE**	21.24 ± 2.71 *	53.07 ± 0.54 *	0.014 ± 0.0081 **
**ASO**	22.30 ± 3.81 *	57.50 ± 1.85 **	0.028 ± 0.012 ***
**Ref**	23.61 ± 0.99 **	69.61 ± 1.40 ***	0.037 ± 0.0003 ***

The enzymatic antioxidant profile: SOD: superoxide dismutase; CAT: catalase; GPx: glutathione peroxidase. Data represent the mean ± SEM for six rats in each group. Statistically significant variations are compared as follows: ASE, ASO and Ref treated groups compared to control group: *, ** and *** indicate significant differences when *p* < 0.05, *p* < 0.01 and *p* < 0.001, respectively.

**Table 4:** Correlation matrix between phytochemicals, oxidative stress markers, fibrinogen and wound reduction.

		TBARS		CD		AOPP		CP		SOD		CAT		GPx		Fibrinogen		Wound Reduction	
	Parameters & Groups	ASE	ASO	ASE	ASO	ASE	ASO	ASE	ASE	ASO	ASO	ASE	ASO	ASE	ASO	ASE	ASO	ASE	ASO
**Extract of AS**	**Polyphenols**	−0.940	−0.140	0.824	0.037	0.544	−0.323	−0.762	−0.762	0.144	0.999 *	−0.762	0.144	−0.961	0.999 *	0.16	−0.55	−0.28	−0.87
	**Flavonoids**	0.630	0.981	−0.806	0.932	0.615	1.000 *	−0.362	−0.362	−0.982	−0.296	−0.362	−0.982	0.053	−0.296	−0.98	−0.60	−0.82	0.76
	**Tannins**	−0.174	0.787	−0.079	0.884	0.999 *	0.658	−0.942	−0.942	−0.785	0.528	−0.942	−0.785	−0.720	0.528	−0.78	−0.99 *	−0.97	0.00
**Oil of AS**	**Polyphenols**	−0.859	0.049	0.702	0.225	0.693	−0.138	−0.871	−0.871	−0.045	0.988	−0.871	−0.045	−0.996	0.988	−0.03	−0.70	−0.45	−0.76
	**Flavonoids**	−0.940	−0.140	0.824	0.037	0.544	−0.323	−0.762	−0.762	0.144	0.999 *	−0.762	0.144	−0.961	0.999 *	0.16	−0.55	−0.28	−0.87

Data represent the values obtained by Pearson correlation analysis. * indicate significant differences when *p* < 0.05.

## Data Availability

All data generated or analyzed during this study are included in this article.

## Supplementary Materials

The following are available online. Figure S1. Calibration curves of total phenolic components, flavonoids and tannins. OD: Optical density.

## Sample Availability

Samples of the compounds are available from the authors.
