# Correction: Saoudi et al. The Role of *Allium subhirsutum* L. in the Attenuation of Dermal Wounds by Modulating Oxidative Stress and Inflammation in *Wistar* Albino Rats. *Molecules* 2021, *26*, 4875

**DOI:** 10.3390/molecules27165332

**Published:** 2022-08-22

**Authors:** Mongi Saoudi, Riadh Badraoui, Ahlem Chira, Mohd Saeed, Nouha Bouali, Salem Elkahoui, Jahoor M. Alam, Choumous Kallel, Abdelfattah El Feki

**Affiliations:** 1 Animal Ecophysiology Laboratory, Sciences Faculty of Sfax, University of Sfax, Sfax 3054, Tunisia; 2 Laboratory of General Biology, Department of Biology, University of Ha’il, Ha’il 81451, Saudi Arabia; 3 Section of Histology and Cytology, Medicine Faculty of Tunis, University of Tunis El Manar, La Rabta, Tunis 1007, Tunisia; 4 Hematology Laboratory, Hospital Habib Bourguiba, Sfax 3029, Tunisia

The authors wish to make the following change to their paper [1]. We realized that, unfortunately, the selection of the corresponding photos for the different groups in Figure 1 was not completely correct. Therefore, after careful verifications, the authors want to publish a corrected version of Figure 1.

The authors apologize for any inconvenience caused and state that the change does not affect the results of the study and the conclusions drawn from it. The original publication has also been updated.

## Figures and Tables

**Figure 1:**
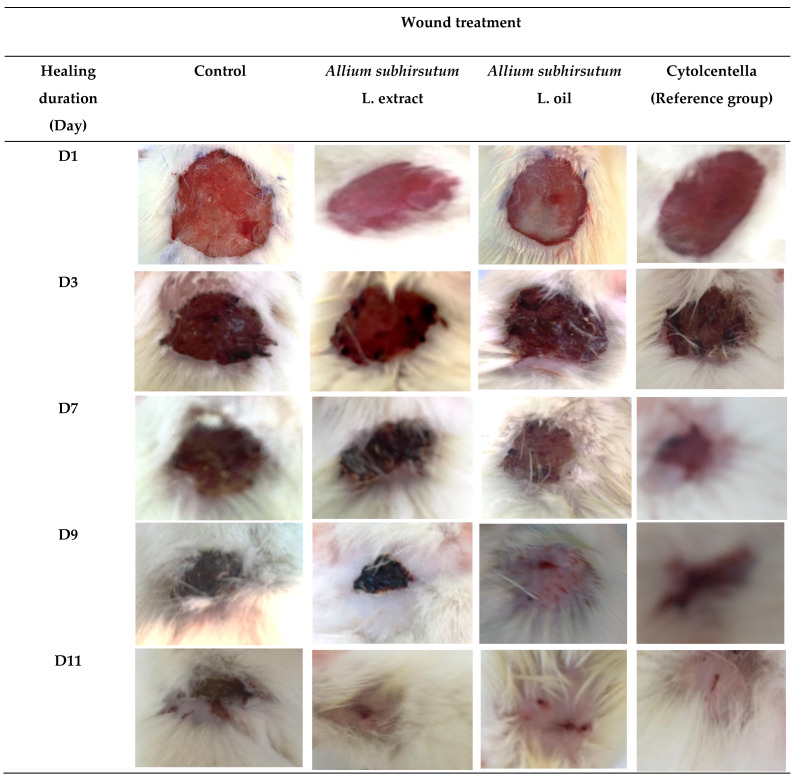
Photographic illustrations of wound healing process in the different experimental groups on 1, 3, 7, 9 and 11 days post-wounding.
