# Supplementary material for: The Role of Allium subhirsutum L. in the Attenuation of Dermal Wounds by Modulating Oxidative Stress and Inflammation in Wistar Albino Rats
Source: Molecules. 2021 Aug 12;26(16):4875. doi: 10.3390/molecules26164875 (PMC8398921; doi:10.3390/molecules26164875)
Supplement: Supplementary file 1 [file molecules-26-04875-s001.zip › molecules-1308028-supplementary.pdf]

Article

The role of *Allium subhirsutum* L. in the attenuation of dermal wounds by modulating oxidative stress and inflammation in *Wistar* albino rats

Mongi Saoudi[1,*], Riadh Badraoui[2,3,*], Ahlem Chira[1], Mohd Saeed[2], Nouha Bouali[2], Salem Elkahoui[2], Jahoor M. Alam[2], Choumous Kallel[4], Abdelfattah ElFeki[1]

1. Animal Ecophysiology Laboratory, Sciences Faculty of Sfax, University of Sfax, Tunisia;mongifss@yahoo.fr (M.S.); chiraahlem@gmail.com (A.C.); abdelfattahelfeki@fss.rnu.tn (A.E.F.)
2. Laboratory of General Biology, Department of Biology, University of Ha'il, Ha'il 81451, Saudi Arabia;mo.saeed@uoh.edu.sa (M.Sae.);nouha_bmail@yahoo.fr (N.B.); s.elkahoui@uoh.edu.sa (S.E.); j.alam@uoh.edu.sa(J.M.A.)
3. Section of Histology and Cytology, Medicine Faculty of Tunis, University of Tunis El Manar, 1007 La Rabta–Tunis, Tunisia; riadh.badraoui@fmt.utm.tn (R.B.)
4. Hematology Laboratory, Hospital Habib Bourguiba, Sfax, Tunisia; kallelC@yahoo.fr (C.K.)

* Correspondence: mongifss@yahoo.fr; Tel.: (+21699740205)

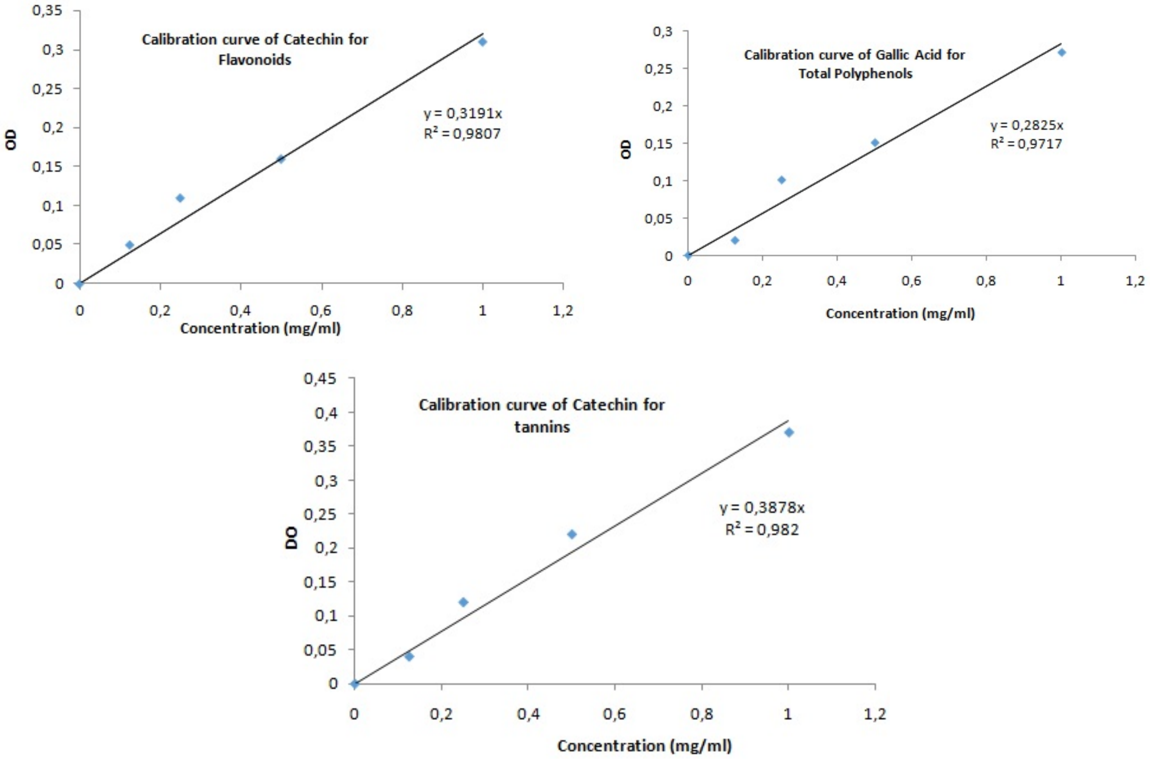

Figure S1. Calibration curves of total phenolic components, flavonoids and tannins. OD: Optical density.
